# Nomogram for individualized prediction of incident multidrug-resistant tuberculosis after completing pulmonary tuberculosis treatment

**DOI:** 10.1038/s41598-020-70748-x

**Published:** 2020-08-13

**Authors:** Qinglin Cheng, Gang Zhao, Xuchu Wang, Le Wang, Min Lu, Qingchun Li, Yifei Wu, Yinyan Huang, Qingjun Jia, Li Xie

**Affiliations:** 1 Division of Infectious Diseases, Hangzhou Center for Disease Control and Prevention, 568 Mingshi Road, Hangzhou, 310021 China; 2 Department of Adolescents and Children Health, School of Public Health, Medical College of Soochow University, Suzhou, 215123 China

**Keywords:** Diseases, Health care, Risk factors

## Abstract

The purposes of this study were to construct a comprehensive nomogram for providing a simple, precise and personalized prediction of incident multidrug-resistant tuberculosis (MDR-TB) after completing pulmonary tuberculosis treatment (CPTBT). A matched case–control study (1:2 ratios) was performed between 2005 and 2018. A multivariable Cox regression analysis was used to evaluate independent predictors of incident MDR-TB after the CPTBT. A comprehensive nomogram was developed based on the multivariable Cox model. Overall, 1, 836 participants were included in this study. We developed and validated a simple-to-use nomogram that predicted the individualized risk of incident MDR-TB by using 10 parameters after the CPTBT. The concordance index of this nomogram was 0.833 [95% confidence interval (CI) 0.807–0.859] and 0.871 (95% CI 0.773–0.969) for the training and validation sets, respectively, which indicated adequate discriminatory power. The calibration curves for the risk of incident MDR-TB showed an optimal agreement between nomogram prediction and actual observation in the training and validation sets, respectively. The high sensitivity and specificity of nomogram was indicated by using a receiver operating characteristic curve analysis. Through this clinic tool, TB control executives could more precisely monitor, estimate and intervene the risk of incident MDR-TB among individuals with CPTBT.

## Introduction

The prevalence of multidrug-resistant tuberculosis (MDR-TB) is increasing rapidly in the world^1^. According to the latest indication given by the World Health Organization (WHO), there are about 500,000 new cases of drug-resistant TB (DR-TB) (of which 78% have the MDR-TB) worldwide in 2018^2^. With 66,000 cases of MDR/rifampicin-resistant TB, China has the second highest number of cases of this disease worldwide^2^. The MDR-TB remains a serious public health issue globally, causing severely social, familial and economic dysfunctions^1^.

In recent years, the continuous monitoring indicates that some individuals with completing PTB treatment (CPTBT) evolve into the MDR-TB after a definite period of time. According to our investigation, we find that PTB patients’ surveillance and management are insufficient after the treatment was completed. Although several studies have revealed that a number of clinical and environmental factors (such as acquired infections, prior irregular treatment, and inadequate treatment management of TB) may affect the prevalence of MDR-TB in TB patients^3–6^, risk factors of incident MDR-TB are not yet fully understood among individuals with CPTBT.

To reduce the morbidity and mortality of MDR-TB, it is urgent that the government and researchers take measures to explore preventive strategies of MDR-TB risk among individuals with CPTBT. Recently investigators have proved the significance of early prediction and assessment on the MDR-TB risk^7,8^. A white paper on the predictive, preventive and personalized medicine^9^ suggests that a central component of preventive strategies is the identification of individuals at risk for development of a disease. Although previous studies have established several models based on predicting the outcome of TB infection and showed certain application value^10,11^, there is currently no model available for the prediction and assessment of MDR-TB risk in individuals with CPTBT.

To date, in the research field of MDR-TB control, though some variables, such as sociodemographic, clinical, and microbiological predictors^12–14^, have been well recognized as determinants of incident MDR-TB in TB patients, few studies focused on the status of incident MDR-TB among individuals with CPTBT, let alone integrated them so as to comprehensively assess a patient’s specific risk of incident MDR-TB. It is now well established from a variety of studies, that the nomogram model is a graphic algorithm tool aimed at providing an approximate computation of a function^15^. In clinical practice, the nomogram has been identified as a practical tool of preventive interventions^16^. In addition, a nomogram can predict and estimate the individualized risk of a disease and quantitatively demonstrate a personalized probability for predicting the incidence of disease outcome^15^.

In the present study, based on a matched case–control study (1:2 ratios), we selected a population with CPTBT as participants and mainly aimed to (a) identify predictors of incident MDR-TB in individuals with CPTBT, hoping to reduce the morbidity and mortality of MDR-TB; and (b) construct a comprehensive nomogram for providing a simple, precise and personalized prediction of incident MDR-TB among individuals with CPTBT.

## Materials and methods

### Sample size calculation

To calculate the sample size, we used the following formula^17^:

$$\begin{aligned} N & = \left[ {Z_{\alpha } \sqrt {\left( {1 + \frac{1}{r}} \right)\overline{p } \left( {1 - \overline{p}} \right)} + {Z_\beta} \sqrt {p_{1} \frac{{\left( {1 - p_{1} } \right)}}{r} + p_{0 } \left( {1 - p_{0} } \right)} { }} \right]^{2} /(p_{1} - p_{0} )^{2} \\ p_{1} & = \frac{{OR \times p_{0} }}{{1 - p_{0} + OR \times p_{0} }} \\ \overline{p } & = \left( {p_{1} + rp_{0} } \right)/\left( {1 + r} \right) \\ \end{aligned}$$

where $N$ = sample size; α = alpha (expected significant level, two-tailed test); β = 1 − power (expected power, two-tailed test); *Z* statistic (*Z*)—*Z* statistic for confidence level; *r*—number of control subjects matched to each case subject; ${p}_{1}$—probability of exposure in the case group; ${p}_{0}$—probability of exposure in the control group (${p}_{0}$ can be estimated as the population prevalence of exposure); *OR* = odds ratio (odds ratio of exposures between cases and controls; *OR* can be estimated as the population *OR* of exposure).

In this study, the investigators present their results with 95% confidence interval (CI), ${Z}_{0.05}$= 1.96 (α = 0.05), ${Z}_{0.10}$= 1.64 (β = 0.10), *r* = 2, ${p}_{0}$= 8.0%^18^, and *OR* = 2.0^19^. In addition, adopting the 'all-comers' design^20^ and considering the loss of follow-up, participants’ rejection rate, and sampling error, the final sample size was determined to be 1,900 in the training set. The validation set was chosen by using an ‘all comers’ design.

### Workflow

**Figure 1:**
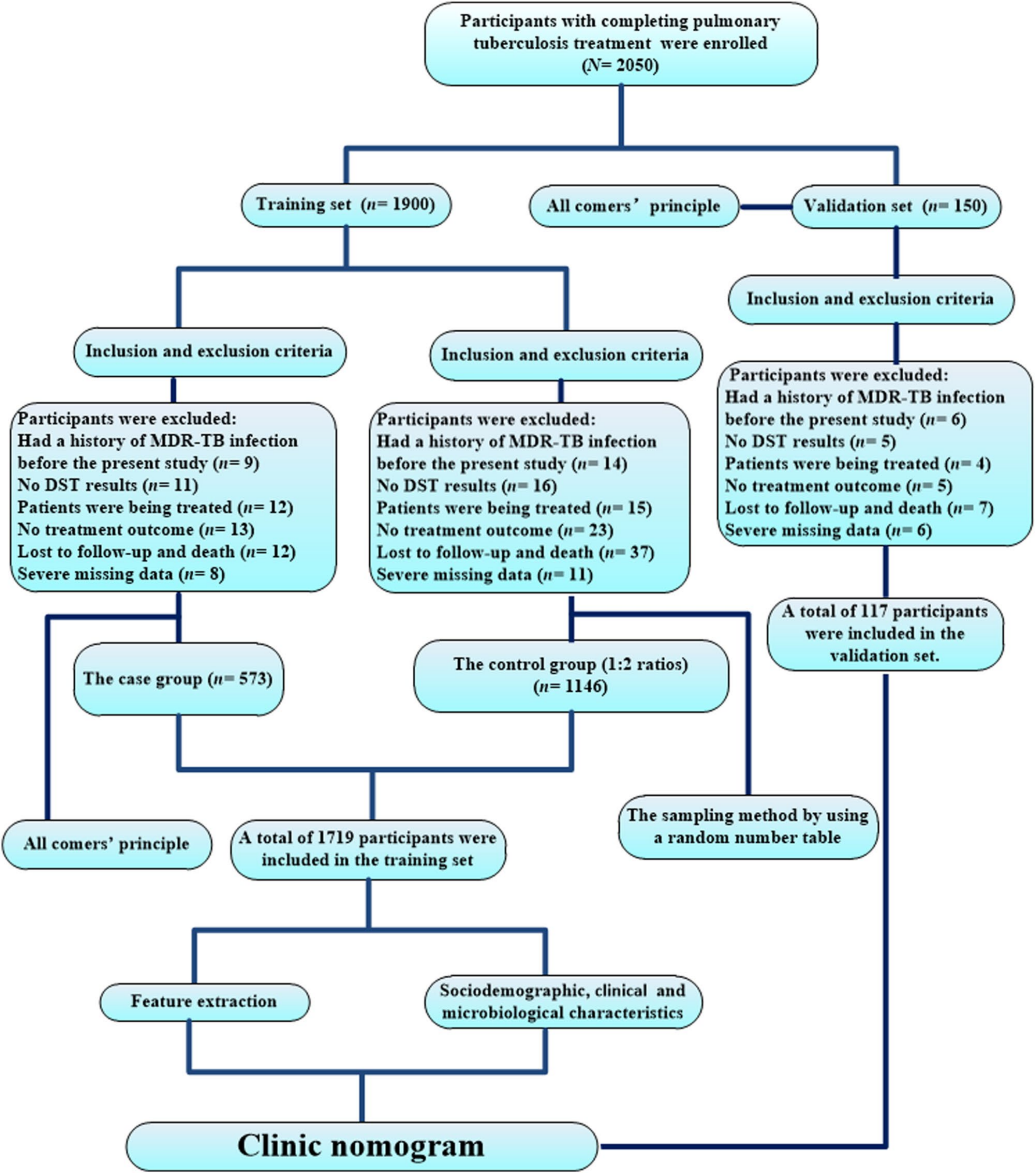
Workflow in this study. MDR-TB: multidrug-resistant tuberculosis; DST: drug susceptibility testing.

This study workflow was summarized in Fig. 1. Two separate datasets were used to develop and validate a risk-prediction tool based on predictors of incident MDR-TB in individuals with CPTBT. Data of a matched case–control study (1:2 ratios) from January 1, 2005 to December 31, 2018 (*n* = 1719) were used to derive the risk of MDR-TB among individuals with CPTBT (i.e., a training dataset), while data from the National TB Surveillance System (NTSS) between January 1 and September 30, 2019 (*n* = 117) was used as an independent dataset to validate the prediction tool (i.e., a validation dataset).

Univariate and multivariate Cox proportional hazards regression models were applied to select optimal risk factors to build a practical instrument for predicting the risk of incident MDR-TB among individuals with CPTBT.

### Study design and settings

This study was based on individuals with CPTBT from 2005 to 2019. A matched case–control study (1:2 ratios) was conducted in Hangzhou, China. The subjects with drug resistance detection who were enrolled in the training set between January 1, 2005 and December 31, 2018 constituted the case–control study. Furthermore, participants with drug resistance detection were enrolled in the validation set between January 1 and September 30, 2019 from an ‘all comers’ approach^20^.

For the present study, the MDR-TB cases were selected from all of TB designated hospitals in Hangzhou City and diagnosed by clinicians through Gene Xpert and traditional drug susceptibility testing (DST)^21^. The cases were selected in this study using the ‘all comers’ principle^20^, as long as they met inclusion and exclusion criteria, and the controls were selected by using a random sampling method from the same TB designated hospitals. In this study, the subjects were classified into ‘incident MDR-TB’ (i.e., the case group) and ‘non-incident MDR-TB’ groups (such as the control group) according to the ultimate outcome. The subjects were ultimately selected if they (a) had a history of PTB but did not have MDR-TB confirmed during their previous treatment episodes; (b) were surviving during the study; (c) had a history of TB treatment; (d) had a definite treatment outcome; and (e) could be followed up. The subjects were excluded if (a) they had a history of MDR-TB infection before the present study; (b) no DST results were reported; (c) TB patients were being treated (i.e., patients with an anti-TB drug therapy during the course of study); (d) no treatment outcome could be obtained; (e) subjects who lost or died during the follow-up visit; and (f) the missing data was severe (Fig. 1).

The starting date of previous anti-TB treatment was defined as the starting time of the observation study; while we defined a patient’s observation ending date as the end date of the study, which was the data of incident MDR-TB or December 31, 2018 in the training set or September 30, 2019 in the validation set. Incident MDR-TB for all years were collected between January 1, 2005, and September 30, 2019. In this study, treatment regimens (TRs) were formulated on the basis of patients’ TB history.

### Data collection

All data in this retrospective observational study were collected from self-designed standard questionnaires and the NTSS, and were entered in duplicate into an electronic database. A self-designed standard questionnaire was used to collect patients’ sociodemographic data. The NTSS was established in 2005 and used to collect patients’ clinical and laboratory test data in our study. Sociodemographic data included age, gender, areas of residence, a history of direct contact, nationality, family income (FI), occupational risk, education levels, and registered household. Clinical data included mode of TB case finding (MCF), associated with TB at other sites, human immunodeficiency virus (HIV) infection, patients with severe infection, comorbidities, different CPTBT including completing newly diagnosed PTB treatment (CNDT) and completing re-treated PTB treatment (CRT), mode of TB case management (MCM), treatment outcomes of previous PTB, time from illness onset to the first medical visit (TIOFMV) and laboratory confirmation (TIOLC), PTB treatment time, the status of using TRs, and chest radiological findings. Laboratory test data included sputum smear, culture, and DST results at baseline and follow-up visits.

Standard participant reporting included sociodemographic, clinical and microbiological information along with initial and follow-up visits. The sociodemographic, clinical and microbiological data of each participant were collected by trained investigators.

### Variables and definitions

The case definitions and classifications used in the present study were consistent with the WHO revised TB definitions and reporting framework^22^. The main outcome variable was measured as incident MDR-TB or non-incident MDR-TB. Table 1 showed the definitions of this study. The main covariate variables were defined and classified based on the WHO and national guidelines^22,23^. Sputum smear, culture, and DST results were defined according to the WHO guideline^22,24^.

**Table 1:** Definitions of this study.

Variables	Definitions
MDR-TB case	A patient infected with TB resistant to at least H and R
Successful treatment	It is defined as follows: (1) previous PTB patients with sputum positive (such as smear-positive, Xpert-positive, and culture-positive) are cured (i.e., patients are with a negative result from the sputum examination) after a course of treatment; (2) previous PTB patients with sputum negative (i.e., smear-negative, Xpert-negative, and culture-negative) have completed a treatment course and showed a significant improvement on the typical pathology of a chest X-ray after a course of treatment
Unsuccessful treatment	Refers to previous PTB cases who are not cured or have not significantly improved on the typical pathology of a chest X-ray after a course of treatment, or have not completed a treatment course (such as patients with loss to follow-up and discontinued therapy)
RPTPs	Including initial treatment failure patients (i.e., during a treatment course, NDPPs with sputum positive are still the sputum examination with a positive result at the end of the 5th month or after a course of treatment), relapse patients (i.e., the PTB recrudesces after NDPPs are cured or have completed a treatment course), returned patients (i.e., re-entry after the abandonment treatment), chronic patients, and other (such as loss to follow-up, discontinued therapy, and unknown or undocumented treatment outcome) patients
CPTBT	After a period of anti-TB treatment, the treatment result of a patient is represented as the successful treatment or unsuccessful treatment
Individuals with CPTBT	Including individuals with completing NDPPs treatment (CNDT) and individuals with completing RPTPs treatment (CRT)
Incident MDR-TB	An MDR-TB case is confirmed from previous PTB treatment starting to the end date of the study
Non-incident MDR-TB	The status of MDR-TB has not happened from previous PTB treatment starting to the end date of the study
Low-income level	The economy income of a family (i.e., below middle-income level) is less than RMB 150,000 Yuan during a year
Middle level and above income	The economy income of a family is more than or equal to RMB 150,000 Yuan during a year
High-risk occupation	Including migrant worker, worker, jobless and vagrant persons
Non-high-risk occupation	Including farmers, teacher, pupils, business services, nurses and nannies, waiters, business services, hospital staffs, herdsmen, fisherman, seafarers and long-distance drivers, official staffs, and being retired
TB case finding	The TB screening (i.e., early finding suspected or confirmed cases) is performed through active modes (i.e., initiatively clinical consultation, recommend based on symptoms, and referral and tracing of PTB suspects reported) and passive modes (such as physical examination, contact examination, and differential diagnosis of other diseases) according to results of the symptom monitoring or chest-X-ray or chest computed tomography or laboratory examination
Standardized treatment course of TB cases	Including 6-month course of NDPPs treatment and 8-month course of RPTPs treatment (i.e., standardized treatment time)
Lost to follow-up	Treatment interrupted for at least two consecutive months
A history of direct contact	Direct contact with MDR-TB patients during the 3 months before illness onset
Frequencies of chest X-ray examination (FCXE)	Refers to the frequency of chest X-ray examination (i.e., greater than 4 times are classified as excellent; the moderate FCXE is defined as the frequency between 3 and 4 times; less than 3 times are classified as poor) during a course of treatment and after the CPTBT. For example, PTB patients usually need to be followed up by the chest X-ray scanning for 4 times during a course of treatment, and be followed up once a year by the chest X-ray scanning after the CPTBT in China
2HRZE/4HR	NDPPs are started on first-line drug therapy consisting of 2 months of R, H, E and Z, and followed by 4 months of R and H; the dosing frequency of TB treatment is a daily dosing throughout therapy
FDC-2HRZE/4HR	NDPPs are started on first-line drug therapy consisting of 2 months of R, H, E and Z, and followed by 4 months of R and H; the dosing frequency of TB treatment is a daily dosing throughout therapy; FDC formulations were used
2H3R3Z3/4H3R3	NDPPs are started on first-line drug therapy consisting of 2 months of R, H and Z, and followed by 4 months of R and H; the dosing frequency of TB treatment is three-times-weekly dosing throughout therapy
2H3R3Z3E3/4H3R3	NDPPs are started on first-line drug therapy consisting of 2 months of R, H, E and Z, and followed by 4 months of R and H; the dosing frequency of TB treatment is three-times-weekly dosing throughout therapy
2HREZ/4H3R3	NDPPs are started on first-line drug therapy consisting of 2 months of R, H, E and Z, and followed by 4 months of R and H; new PTB patients may receive a daily intensive phase followed by a three-times weekly continuation phase
2HRZES/6HRE	RPTPs without R resistance detected on Xpert are started on WHO guidelines, i.e., 2 months of R, H, E, Z and S, and followed by 6 months of R, H and E; the dosing frequency of TB treatment is a daily dosing throughout therapy
3HRZE/6HRE	RPTPs without R resistance detected on Xpert are started on WHO guidelines, i.e., 3 months of R, H, E and Z, and followed by 6 months of R, H and E; the dosing frequency of TB treatment is a daily dosing throughout therapy
3HRZES/6HRE	RPTPs without R resistance detected on Xpert are started on WHO guidelines, i.e., 3 months of R, H, E, Z and S, and followed by 6 months of R, H and E; the dosing frequency of TB treatment is a daily dosing throughout therapy
2H3R3Z3E3S3/6H3R3E3	RPTPs without R resistance detected on Xpert are started on WHO guidelines, i.e., 2 months of R, H, E, Z and S, and followed by 6 months of R, H and E; the dosing frequency of TB treatment is three-times-weekly dosing throughout therapy
Individualized treatment regimens	Clinic doctors develop the individualized therapeutic schedule including 4–6 drugs (typically including a fluoroquinolone and/or injectable secondline drug and three or four first-line drugs therapy) based on clinical experience

MDR-TB: multidrug-resistant tuberculosis; TB: tuberculosis; PTB: pulmonary tuberculosis; CPTBT: completing PTB treatment; NDPPs: newly diagnosed pulmonary TB patients; RPTPs: Re-treated pulmonary TB patients; CNDT: completing NDPPs treatment; CRT: completing RPTPs treatment; FDC: fixed-dose combination; H: isoniazid; R: rifampicin; Z: pyrazinamide; E: ethambutol; S: streptomycin; WHO: World Health Organization.

### Laboratory methods

Traditional laboratory test methods (such as sputum smear and culture) were mainly used for the diagnosis of TB from 2005 to 2014 in the present study^25^. Moreover, methods of TB diagnosis mainly included the molecular biological detection (e.g., a Gene Xpert method) and traditional laboratory test between 2015 and 2019^20^. A case of MDR-TB was confirmed by using the DST in the TB designated laboratory. The DST was performed on all culture positive isolates against first line [isoniazid (H), rifampicin (R), pyrazinamide (Z), ethambutol (E) and streptomycin (S)] and second line anti-TB drugs (kanamycin and ofloxacin)^24^. The methods of DST usually include the conventional microbiological DST and Gene Xpert Mycobacterium TB (MTB)/R. The conventional microbiological DST is performed using solid or automated liquid culture media system (BACTEC MGIT 960; Becton Dickinson, Sparks, Maryland, USA) according to standard procedures^24^. The method of Gene Xpert MTB/R denotes that the resistance to R is detected by using Gene technology^24^.

The DST detection are performed during the course of follow-up visits. For conventional microbiological DST and Gene Xpert MTB/R detections, samples collected are sent to the TB Program Laboratory of Hangzhou Center for Disease Control and Prevention (a biosafety level-3 laboratory with proficiency testing approved by National Reference Laboratory in China). MDR-TB cases of laboratory cross-contamination are excluded. Drugs with borderline resistance are considered to be resistant.

### Statistical analysis

Outcome variable was categorized as a binary variable with incident MDR-TB and non-incident MDR-TB categories. Descriptive analyses were used to examine the distribution of characteristics of participants in the training and validation sets. Continuous variables were described by using mean with standard deviation while categorical data was analyzed by using percent (proportion). A Pearson Chi-square test was used for categorical variables and an independent sample *t*-test for continuous variables in both training and validation sets.

We used univariable and multivariable Cox regression models to analyze the risk of incident MDR-TB among individuals with CPTBT. Patients who died, loss to follow-up visit and could not be evaluated were excluded from the analysis. Univariable Cox proportional hazard regression analysis was conducted to determine factors associated with incident MDR-TB. Variables were analyzed using hazard ratio (HR) generated by univariable Cox proportional hazards regression.

Subsequently, independent predictors associated with incident MDR-TB were evaluated using HR generated by a multivariable Cox proportional hazard regression model. All variables with *P* value of ≤ 0.05 were included into a multivariable Cox proportional hazard regression model using backward stepwise method based on the minimum statistics of the Akaike information criterion. Variables with *P* value of < 0.05 were considered statistically significant in the multivariable Cox proportional hazard regression model and were included in the final predictive model.

Based on the results of multivariate Cox regression analysis in the training set, a nomogram was developed and validated. Nomogram validation included two components. First, the internal validation of clinic nomogram was performed using a concordance index (C-index) by subjecting the nomogram to bootstrapping with 200 resamples^26^. The predictive accuracy of 1-, 5-, and 10-year probability of incident MDR-TB was evaluated by using the area under receiver operating characteristic (ROC) curve (AUC). Next, the calibration of nomogram was performed by comparing the predicted probability of incident MDR-TB with the observed probability of incident MDR-TB after bias correction (i.e., using a calibration curve). In addition, for external validation, we predicted the risk of incident MDR-TB using data from the other 117 individuals of validation set.

All statistical analyses were performed with R software (version i 386 3.6.1; www.R-project.org, 2019). The multivariable Cox proportional hazard regression model was created using the R software’s ‘survival’ package, while the nomogram and calibration curves were plotted using the ‘rms’ package.

### Ethics approval and consent to participate

The study protocol was approved by the Hangzhou Center for Disease Control and Prevention Ethics Committee. Written informed consent was obtained from all participants, or from guardians or parents on behalf of participants under the age of 18 years. In addition, all methods were carried out in accordance with relevant guidelines and regulations.

## Results

### Characteristics of the subjects

A flow diagram summarizing the identified eligible subjects and the study participants was shown in Fig. 1. Baseline characteristics of the study population were listed in Table 2.

**Table 2:** Baseline characteristics of the study population (*N* = 1836).

Variables	Training set (*n* = 1719)	Validation set (*n* = 117)	*P* value
**Age (mean ± SD, years)**	48.90 ± 20.95	49.41 ± 21.84	0.799
**Gender**			
Male	1,216 (70.74)	88 (75.21)	0.302
Female	503 (29.26)	29 (24.79)	
**Nationality**			
Han	1,700 (98.89)	114 (97.44)	0.161
National minority	19 (1.11)	3 (2.56)	
**Occupational risk**			
High-risk	362 (21.06)	33 (28.21)	0.069
Non-high-risk	1,357 (78.94)	84 (71.79)	
**Education levels**			
High school and below	1,270 (73.88)	87 (74.36)	0.909
Universities and higher	449 (26.12)	30 (25.64)	
**Residences**			
Rural areas	554 (32.23)	36 (30.77)	0.744
Urban areas	1,165 (67.77)	81 (69.23)	
**Registered household**			
Migrant individuals with CPTBT	768 (44.68)	50 (42.74)	0.683
Resident individuals with CPTBT	951 (55.32)	67 (57.26)	
**Family income**			
Low level	558 (32.46)	48 (41.03)	0.057
Middle level and above	1,161 (67.54)	69 (58.97)	
**Types of MDR-TB diagnosis**			
Traditional susceptibility test	1,212 (70.51)	81 (69.23)	0.770
Gene Xpert MTB/R	507 (29.49)	36 (30.77)	
**Different individuals with CPTBT**			
CNDT	1,411 (82.08)	97 (82.91)	0.822
CRT	308 (17.92)	20 (17.09)	
**Outcomes of previous TB treatment**			
Unsuccessful treatment	257 (14.95)	21 (17.95)	0.381
Successful treatment	1,462 (85.05)	96 (82.05)	

Data are presented as No. (%), unless otherwise stated.

TB: tuberculosis; MDR-TB: multidrug-resistant tuberculosis; CPTBT: completing pulmonary TB treatment; CNDT: completing newly diagnosed pulmonary TB treatment; CRT: completing re-treated pulmonary TB treatment; SD: standard deviation; MTB: *mycobacterium tuberculosis*; R: rifampicin.

We retrospectively studied 1,836 subjects with CPTBT in Hangzhou from January 1, 2005 to September 30, 2019. Participants in the training set (*n* = 1719) and the external validation set (*n* = 117) were analyzed respectively. There was not a significant difference between the two sets (Table 2). The mean age was 48.90 ± 20.95 and 49.41 ± 21.84, and the ratio of males to females was 2.42 to 1 and 3.03 to 1 in the training and validation sets, respectively. Notably, most of the subjects [1, 357 (73.91%)] were with the education level of high school and below (Table 2).

### Predictors’ selection

Table 3 summarized the results of the univariate analyses of the association between an individual covariate and the risk of incident MDR-TB among individuals with CPTBT. Twenty of the 44 tested covariates were associated with a high risk of incident MDR-TB from this study population in the training set (*P* ≤ 0.05). The significant covariates were (a) sociodemographic characteristics, including age < 60 years, a history of direct contact, family income of low level, high-risk occupation, high school and below, and rural areas, (b) clinical characteristics, including passive MCF, HIV infection, CRT, unsuccessful treatment, TIOFMV, FDC-2HRZE/4HR, 2HRZES/6HRE, 3HRZES/6HRE, excellent frequencies of chest X-ray examination (FCXE), duration of pulmonary cavities (DPC), and duration of abnormal X-ray findings, and (c) microbiological characteristics, including frequencies of sputum culture, duration of positive sputum culture (DPSC), and duration of negative sputum culture. The remaining 24 covariates, including gender, nationality, a history of direct contact (e.g., unknown), registered household, associated with TB at other sites, comorbidities, patients with severe infection, MCM, PTB treatment time, TIOLC, 2H3R3Z3/4H3R3, 2H3R3Z3E3/4H3R3, 2HREZ/4H3R3, 2HRZE/4HR, 3HRZE/6HRE, 2H3R3Z3E3S3/6H3R3E3, individualized TRs [i.e., individualized TRs of newly diagnosed PTB patients (NDPPs) and re-treatment PTB patients (RPTPs)], duration of pulmonary miliary tubercles, duration without radiological findings, duration without sputum culture, frequencies of sputum smear, duration of positive sputum smear, duration of negative sputum smear, and duration without sputum smear, were not associated with incident MDR-TB among individuals with CPTBT (*P* > 0.05).

**Table 3:** Univariate Cox regression model showing risk factors associated with incident MDR-TB in the training and validation sets (*N* = 1836).

Variables	Training set (n = 1719)*			Validation set (n = 117)*		
	No	HR (95% CI)	*P* value	No	HR (95% CI)	*P* value
**Sociodemographic characteristics**						
Age (years)						
< 60	1,108	**1.90 (1.57–2.31)**	**< 0.001**	69	**2.74 (1.26–6.00)**	**0.011**
≥ 60	611	Reference		48	Reference	
Gender						
Male	1,216	1.18 (0.85–1.42)	0.083	88	1.40 (0.64–3.06)	0.399
Female	503	Reference		29	Reference	
Nationality						
Han	1,700	1.03 (0.46–2.31)	0.940	114	0.98 (0.13–7.17)	0.986
National minority	19	Reference		3	Reference	
A history of direct contact						
Yes	225	**3.25 (2.96–3.56)**	**< 0.001**	26	**3.56 (2.38–5.33)**	**< 0.001**
Unknown	272	1.18 (0.76–1.84)	0.231	20	0.99 (0.46–1.69)	0.213
No	1,222	Reference		71	Reference	
Family income						
Low-income level	558	**1.33 (1.13–1.58)**	**0.001**	49	1.42 (0.76–2.68)	0.275
Middle level and above	1,161	Reference		68	Reference	
Occupational risk						
High-risk	362	**1.51 (1.26–1.80)**	**< 0.001**	33	**2.22 (1.18–4.18)**	**0.013**
Non-high-risk	1,357	Reference		84	Reference	
Education levels						
High school and below	1,270	**1.26 (1.04–1.54)**	**0.022**	87	1.44 (0.69–3.15)	0.359
Universities and higher	449	Reference		30	Reference	
Residences						
Rural areas	554	**1.32 (1.12–1.57)**	**0.001**	36	1.45 (0.76–2.76)	0.265
Urban areas	1,165	Reference		81	Reference	
Registered household on individuals with CPTBT						
Migrants	768	1.13 (0.88–1.34)	0.143	50	1.29 (0.68–2.45)	0.445
Inhabitants	951	Reference		67	Reference	
**Clinical characteristics**						
Mode of TB case finding						
Passive	418	**2.76 1.59–4.78)**	**< 0.001**	35	**2.13 (1.12–4.05)**	**0.022**
Active	1,301	Reference		82	Reference	
Associated with TB at other sites						
Yes	96	1.09 (0.74–1.61)	0.663	7	0.41 (0.06–3.02)	0.383
No	1,623	Reference		110	Reference	
Comorbidities						
Yes	128	0.80 (0.54–1.16)	0.240	15	0.93 (0.33–2.64)	0.892
No	1,591	Reference		102	Reference	
HIV infection						
Positive	56	**3.96 (2.97–5.26)**	**< 0.001**	9	**2.96 (1.16–7.61)**	**0.024**
Negative	1,663	Reference		108	Reference	
Patients with severe infection						
Yes	124	1.07 (0.80–1.45)	0.660	8	1.20 (0.37–3.91)	0.763
No	1,595	Reference		109	Reference	
Mode of TB case management						
FMSM	236	1.16 (0.93–1.44)	0.197	14	1.19 (0.49–2.89)	0.700
CDM	1,483	Reference		103	Reference	
Different individuals with CPTBT						
CRT	308	**1.67 (1.38–2.02)**	**< 0.001**	43	**3.66 (1.86–7.21)**	**< 0.001**
CNDT	1,411	Reference		74	Reference	
Outcomes of previous TB treatment						
Unsuccessful treatment	257	**5.14 (4.33–6.11)**	**< 0.001**	21	**5.37 (2.54–11.34)**	**< 0.001**
Successful treatment	1,462	Reference		96	Reference	
PTB treatment time (days)	1,719	0.99 (0.99–1.00)	0.130	117	**1.05 (1.02–2.01)**	**0.015**
TIOFMV (days)	1,586	**1.01 (1.00–1.02)**	**0.025**	108	0.99 (0.98–1.01)	0.162
TIOLC (days)	1,719	0.99 (0.99–1.00)	0.945	117	0.99 (0.98–1.01)	0.100
TRs from individuals with CPTBT						
2H3R3Z3/4H3R3	13	0.67 (0.32–1.43)	0.304	11	0.72 (0.22–2.36)	0.592
2H3R3Z3E3/4H3R3	93	0.82 (0.62–1.10)	0.185	6	0.74 (0.22–2.57)	0.640
2HREZ/4H3R3	21	0.85 (0.40–1.79)	0.611	7	0.97 (0.23–4.03)	0.963
2HRZE/4HR	676	0.87 (0.73–1.04)	0.114	40	1.03 (0.97–2.02)	0.596
FDC-2HRZE/4HR	91	**0.57 (0.36–0.93)**	**0.025**	6	0.73 (0.17–3.11)	0.674
2HRZES/6HRE	53	**1.64 (1.12–2.40)**	**0.011**	5	0.48 (0.07–3.51)	0.469
3HRZE/6HRE	83	1.25 (0.89–1.77)	0.196	8	1.84 (0.65–5.21)	0.251
3HRZES/6HRE	43	**4.79 (2.47–9.29)**	**< 0.001**	14	**2.44 (1.11–5.37)**	**0.027**
2H3R3Z3E3S3/6H3R3E3	15	1.00 (0.53–1.87)	0.988	12	1.23 (0.43–3.47)	0.699
Individualized TRs	631	1.11 (0.93–1.33)	0.266	8	1.39 (0.71–2.75)	0.334
**Chest imaging**						
Excellent FCXE	220	**0.90 (0.85–0.96)**	**0.001**	22	1.01 (0.83–1.23)	0.911
Moderate FCXE	1,267	1.19 (0.65–2.94)	0.324	71	1.82 (0.54–2.31)	0.365
Poor FCXE	232	2.03 (0.82–3.25)	0.216	14	2.31 (0.44–3.21)	0.568
DPC (months)	706	**1.21 (1.14–1.28)**	**< 0.001**	38	**1.53 (1.15–2.04)**	**0.004**
Duration of miliary tubercles (months)	15	1.08 (0.54–2.15)	0.836	7	0.77 (0.19–3.21)	0.720
DAF (months)	1,692	**1.15 (1.09–1.21)**	**< 0.001**	109	0.98 (0.78–1.23)	0.852
Duration without findings (months)	27	0.81 (0.53–1.25)	0.337	8	0.72 (0.28–1.84)	0.492
**Microbiological characteristics**						
FSC	1,350	**0.88 (0.83–0.93)**	**< 0.001**	94	**0.77 (0.60–1.00)**	**0.050**
DPSC (months)	954	**1.14 (1.04–1.24)**	**0.004**	66	0.99 (0.64–1.54)	0.968
DNSC (months)	396	**0.77 (0.69–0.86)**	**< 0.001**	28	**0.46 (0.22–0.97)**	**0.042**
DWSC (months)	369	0.92 (0.79–1.06)	0.249	23	1.01 (0.33–3.11)	0.983
FSS	1,699	1.05 (0.95–1.18)	0.434	115	0.94 (0.87–1.02)	0.121
DPSS (months)	607	1.02 (0.98–1.06)	0.405	34	1.05 (0.86–1.28)	0.628
DNSS (months)	1,092	0.98 (0.81–1.20)	0.880	81	**0.89 (0.80–0.99)**	**0.030**
DWSS (months)	20	0.62 (0.32–1.21)	0.161	2	0.83 (0.09–7.71)	0.869

Data are shown as No., hazard ratio (95% CI), and *P* value.

MDR-TB: multidrug-resistant tuberculosis; TB: tuberculosis; PTB: pulmonary tuberculosis; CPTBT: completing pulmonary TB treatment; CNDT: completing newly diagnosed pulmonary TB treatment; CRT: completing re-treated pulmonary TB treatment; HIV: human immunodeficiency virus; HR: hazard ratio; TRs: treatment regimens; FDC: fixed-dose combination; H: isoniazid; R: rifampicin; Z: pyrazinamide; E: ethambutol; S: streptomycin; CI: confidence interval; FMSM: family members' management or self-management; CDM: community doctor management; TIOLC: time from illness onset to laboratory confirmation; MCF: mode of TB case finding; TIOFMV: time from illness onset to the first medical visit; FCXE: frequencies of chest X-ray examination; DPC: duration of pulmonary cavities; DAF: duration of abnormal X-ray findings; FSC: frequencies of sputum culture; DPSC: duration of positive sputum culture; DNSC: duration of negative sputum culture; DWSC: duration without sputum culture; FSS: frequencies of sputum smear; DPSS: duration of positive sputum smear; DNSS: duration of negative sputum smear; DWSS: duration without sputum smear.

*Bold values are those that reach statistical significance (*P* < 0.05).

**Figure 2:**
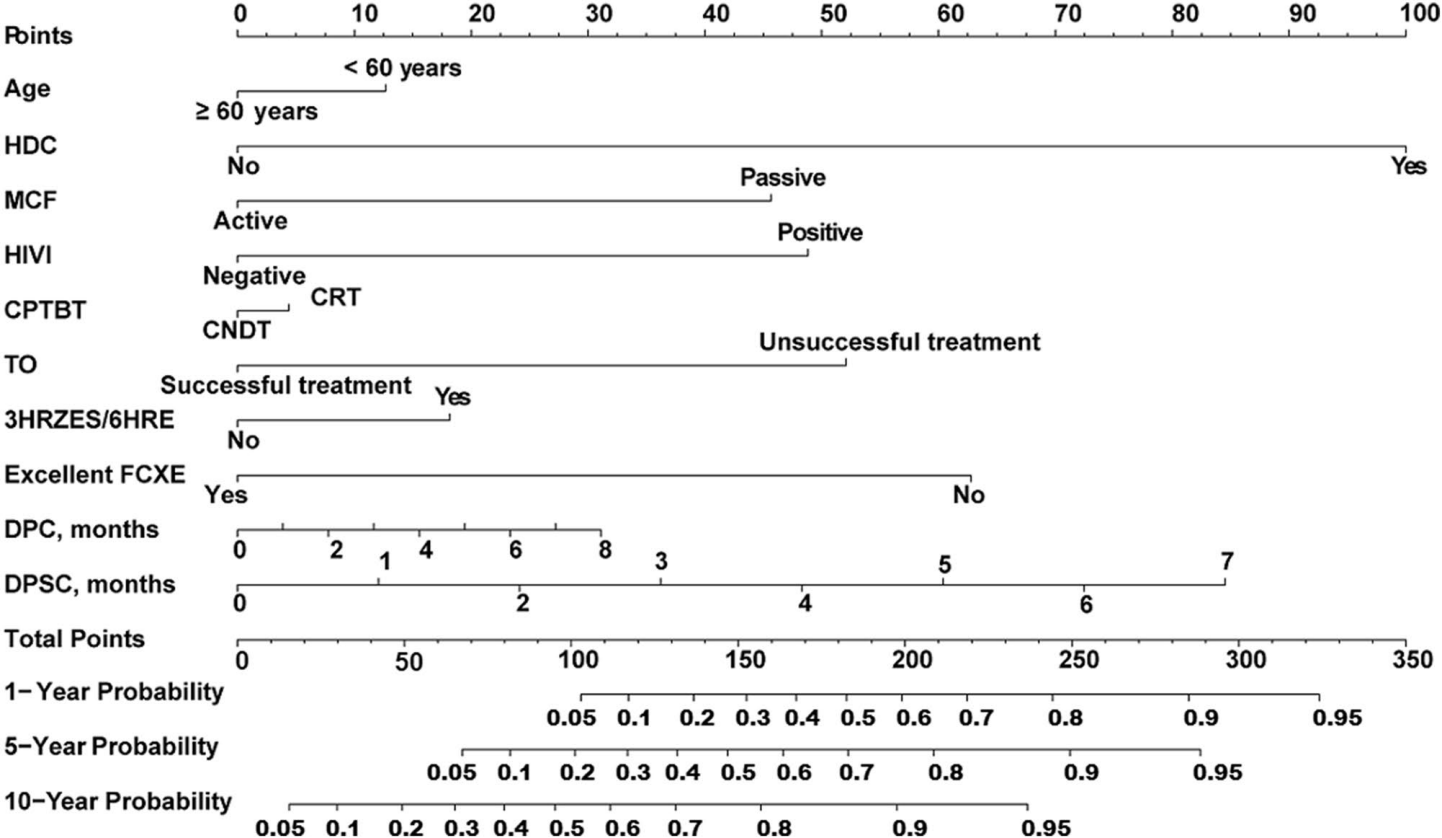
The nomogram for individualized predicting the risk of incident MDR-TB from individuals with CPTBT. MDR-TB: multidrug-resistant tuberculosis; CPTBT: completing pulmonary TB treatment; CNDT: completing newly diagnosed pulmonary TB treatment; CRT: completing re-treated pulmonary TB treatment; HDC: a history of direct contact; MCF: mode of TB case finding; HIVI: human immunodeficiency virus infection; TO: treatment outcome; H: isoniazid; R: rifampicin; Z: pyrazinamide; E: ethambutol; S: streptomycin; FCXE: frequencies of chest X-ray examination; DPC: duration of pulmonary cavities; DPSC: duration of positive sputum culture.

To further explore independent predictors of incident MDR-TB in individuals with CPTBT, we performed a multivariate Cox proportional hazard regression analysis. Table 4 listed the multivariable Cox regression results for this study population. The analysis showed that less than 60 years, a history of direct contact, passive MCF, HIV infection, CRT, unsuccessful treatment, excellent FCXE, 3HRZES/6HRE, DPC, and DPSC were significantly linked to the MDR-TB risk in the training set (*P < *0.05). From this model, we could also see that the unsuccessful treatment (HR 2.72, 95% CI 2.20–3.37, *P < *0.001) was one of the strongest predictors for incident MDR-TB in this population (Table 4). These findings were used to create a practical clinical nomogram for predicting the probability of incident MDR-TB among individuals with CPTBT (Fig. 2).

**Table 4:** Multivariate Cox regression model showing risk factors associated with incident MDR-TB in the training and validation sets (*N* = 1836).

Variables	Training set (*n* = 1719)*			Validation set (*n* = 117)*		
	No	HR (95% CI)	*P* value	No	HR (95% CI)	*P* value
**Sociodemographic characteristics**						
Age < 60 years	1,108	**1.25 (1.01–1.57)**	**0.049**	69	1.37 (0.51–3.71)	0.531
Low-level FI	558	1.05 (0.56–1.99)	0.867	48	NA	NA
High-risk occupation	362	0.98 (0.78–1.22)	0.833	33	1.90 (0.78–4.65)	0.160
High school and below	1,270	1.22 (0.95–1.58)	0.125	87	NA	NA
Rural areas	554	1.13 (0.64–1.97)	0.256	36	NA	NA
A history of direct contact	225	**2.71 (2.42–3.04)**	**< 0.001**	26	**2.34 (1.33–4.13)**	**0.003**
**Clinical characteristics**						
Passive MCF	418	**2.38 (1.24–4.58)**	**0.009**	35	1.28 (0.46–3.53)	0.639
HIV infection	56	**2.36 (1.75–3.18)**	**< 0.001**	9	1.32 (0.42–4.16)	0.640
CRT	308	**1.36 (1.11–1.68)**	**0.004**	43	1.83 (0.26–12.70)	0.540
Unsuccessful treatment	257	**2.72 (2.20–3.37)**	**< 0.001**	21	**2.65 (1.06–6.62)**	**0.037**
PTB treatment time (days)	1,719	NA	NA	117	**1.03 (1.01–1.14)**	**0.016**
TIOFMV (days)	1,586	1.00 (0.99–1.01)	0.368	108	NA	NA
FDC-2HRZE/4HR	91	0.90 (0.52–1.54)	0.692	6	NA	NA
2HRZES/6HRE	53	0.71 (0.47–1.06)	0.090	5	NA	NA
3HRZES/6HRE	43	**2.18 (1.31–3.62)**	**0.003**	14	0.71 (0.26–1.96)	0.510
**Chest imaging**						
Excellent FCXE	220	**0.71 (0.65–0.77)**	**< 0.001**	22	NA	NA
DPC, months	706	**1.18 (1.10–1.27)**	**< 0.001**	38	**1.51 (1.01–2.25)**	**0.046**
DAF, months	1,692	1.21 (0.91–1.34)	0.253	109	NA	NA
**Microbiological characteristics**						
FSC	1,350	1.00 (0.87–1.15)	0.978	94	0.71 (0.45–1.13)	0.148
DPSC, months	954	**1.26 (1.10–1.44)**	**0.001**	66	NA	NA
DNSC, months	396	0.90 (0.75–1.08)	0.997	28	0.95 (0.44–2.05)	0.896

Data are shown as No., hazard ratio (95% CI), and *P* value.

MDR-TB: multidrug-resistant tuberculosis; TB: tuberculosis; PTB: pulmonary tuberculosis; FI: family income; HIV: human immunodeficiency virus; HR: hazard ratio; MCF: mode of TB case finding; CRT: completing re-treated pulmonary TB treatment; TIOFMV: time from illness onset to the first medical visit; FDC: fixed-dose combination; H: isoniazid; R: rifampicin; Z: pyrazinamide; E: ethambutol; S: streptomycin; CI: confidence interval; NA: not available; FCXE: frequencies of chest X-ray examination; DPC: duration of pulmonary cavities; DAF: duration of abnormal X-ray findings; FSC: frequencies of sputum culture; DPSC: duration of positive sputum culture; DNSC: duration of negative sputum culture.

*Bold values are those that reach statistical significance (*P* < 0.05).

### Construction of the nomogram

A nomogram is developed to assess the risk of incident MDR-TB using significant factors from the 1,719 patients’ data in the training set. With 10 independent predictors of training set, it is possible to create a nomogram to predict the probability of incident MDR-TB among individuals with CPTBT (Fig. 2). The top row of the nomogram corresponds to the general score. For each predictor listed on the left (including less than 60 years, a history of direct contact, passive MCF, HIV infection, CRT, unsuccessful treatment, 3HRZES/6HRE, excellent FCXE, DPC, and DPSC), there is a corresponding row on the right indicating possible descriptors. After characterizing the patient for each predictor, a perpendicular line toward the first row should be drawn to identify the value. This action should be performed for all 10 predictors, followed by tallying the final score. This final score should be identified in a total point row and then a perpendicular line is drawn that corresponds to the probability of incident MDR-TB from individuals with CPTBT.

### Calibration and validation of the nomogram

**Figure 3:**
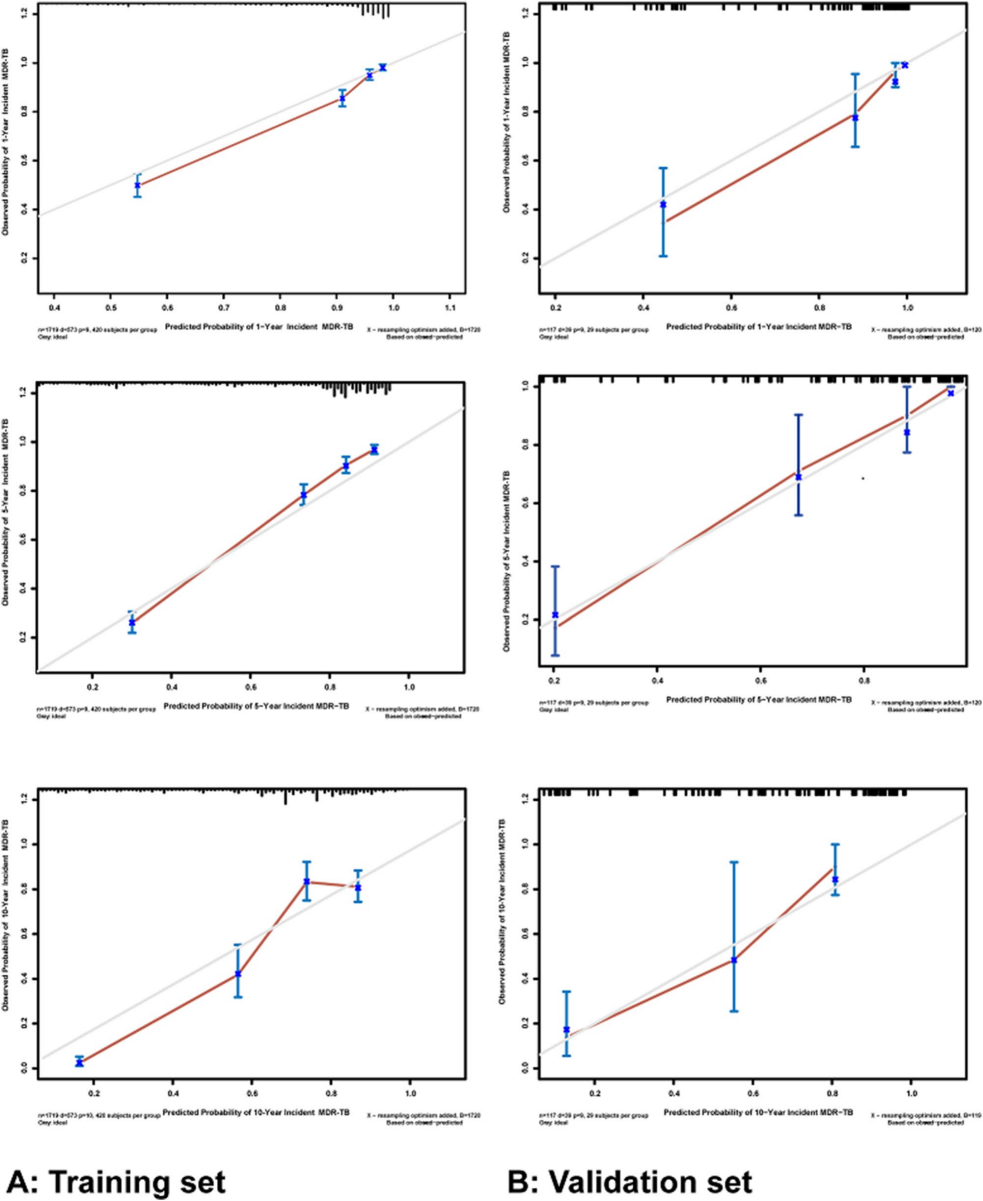
The calibration curves for predicting the risk of incident MDR-TB from individuals with CPTBT at each time point in the training set (**A**) and the external validation set (**B**), respectively. Nomogram predicted the probability of incident MDR-TB from individuals with CPTBT which is plotted on the X-axis and observed the probability of incident MDR-TB from individuals with CPTBT which is plotted on the Y-axis. MDR-TB: multidrug-resistant tuberculosis; CPTBT: completing pulmonary TB treatment.

After internal validation using the bootstrap technique, the C-index of this nomogram is 0.833 (95% CI 0.807–0.859) and 0.871 (95% CI 0.773–0.969) for the training and validation sets, respectively, which indicates adequate discriminatory power. The calibration plots are also performed separately using the training and external validation sets. As shown in Fig. 3A, the calibration plots show that the predicted 1-, 5-, and 10-year probability of incident MDR-TB corresponded closely with the actual 1-, 5-, and 10-year probability of incident MDR-TB estimated in the training set. Figure 3B illustrates that the nomogram appears well calibrated, and there is a strong correlation between predicted and observed outcomes across the spectrum of predictions in the external validation set.

**Figure 4:**
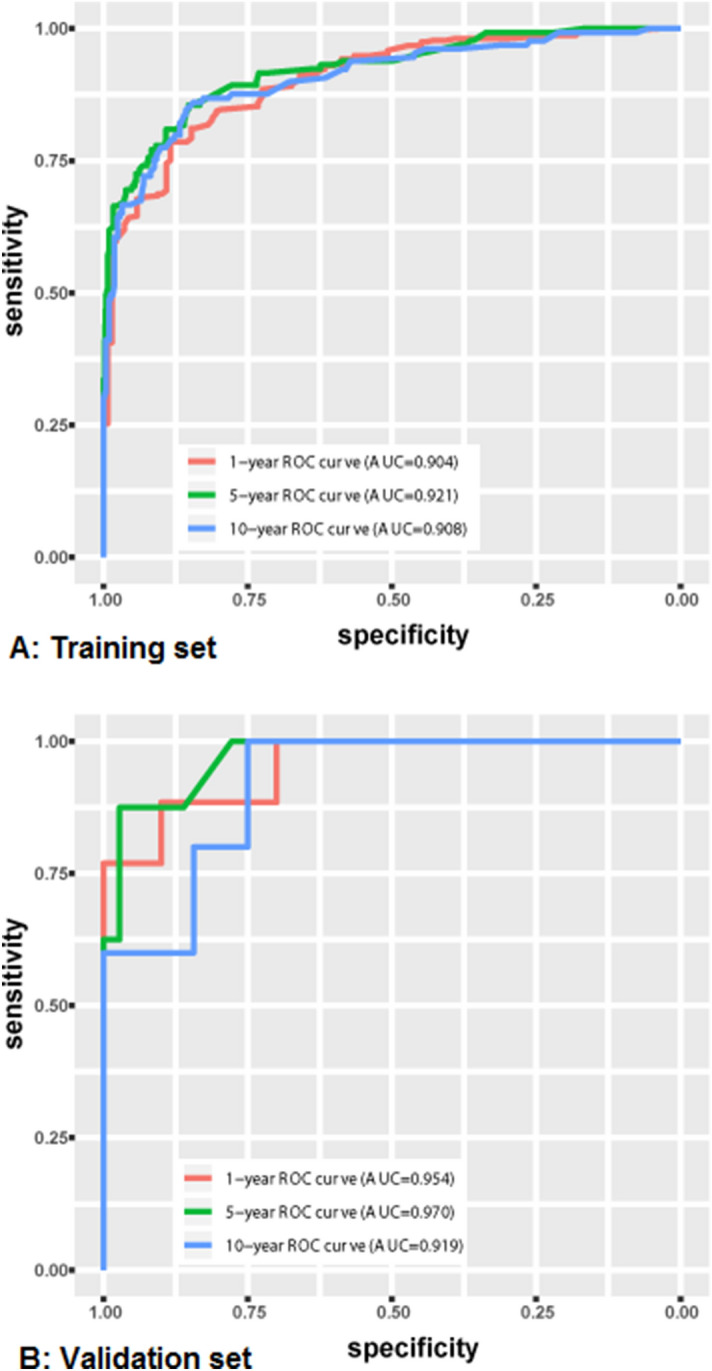
Area under receiver operating characteristic (ROC) curves (AUCs) of the nomogram. The AUCs of the nomogram to predict overall incidence at 1-, 5-, and 10-year (**A**) using the training set as well as at 1-, 5-, and 10-year (**B**) using the external validation set.

For the training set, the AUCs of the nomogram predicting the 1-, 5- and 10-year incidence of MDR-TB are 0.904, 0.921, and 0.908, respectively (Fig. 4A). Regarding the external validation set, the AUCs of the nomogram for predicting the 1-, 5- and 10-year incidence of MDR-TB are 0.954, 0.970, and 0.919, respectively (Fig. 4B). As Fig. 4 shows, the nomogram demonstrates the superior prediction ability of incidence of MDR-TB.

### Predicting an individual’s MDR-TB risk among individuals with CPTBT

To make it easier to interpret our results, we represented the final reduced model with a nomogram that can be used to calculate a prognostic score and estimate the risk of incident MDR-TB for an individual with CPTBT (Fig. 2). The nomogram produced the following mathematical predictive model for the presence of incident MDR-TB risk in the training set, with *h* (*t, x*) denoting the probability of incident MDR-TB among individuals with CPTBT^27^:

$$\begin{aligned} h\left( {t, \, x} \right) & = h_{0} \left( t \right){\exp}\left( {\beta_{{1}} x_{{1}} + \cdots + \beta_{{\text{i}}} x_{{\text{i}}} + \cdots + \beta_{{\text{k}}} x_{{\text{k}}} } \right) \\ & = h_{0} \left( t \right){\exp}[0.{9975} \times ({\text{a history of direct}} \\ & \quad contact) + 0.{2253} \times \left( {{\text{less than 6}}0{\text{ years}}} \right) + 0.0{544} \times {\text{CRT}} + 0.{8685} \\ & \quad \times \left( {\text{passive MCF}} \right) + 0.8596 \times (HIVinfection) + 1.0023 \\ & \quad \times (unsuccessful \, treatment) + 0.7779 \times \left( {3HRZES/6HRE} \right) - 0.{3447} \\ & \quad \times (excellent \, FCXE) + 0.1682 \times DPC + 0.2308 \times DPSC] \\ \end{aligned}$$

where *h* (*t, x*) is the hazard at time *t* after a defined starting point for an individual with variables *x* = (*x*_1_… *x*_i_ … *x*_k_) is being predicted by *h*_0_ (*t*), the so-called underlying hazard at time *t*, and the predictor variables *x*_1_ to *x*_k_ (recorded at time zero), each variable *x*_i_ being multiplied by a corresponding regression coefficient *β*_i_. Here, exp stands for exponential function, e.g., exp (*βx*) = e^*βx*^, and the underlying hazard *h*_0_ (*t*) is the hazard at time *t* of an individual whose *x*_i_’s are all zero.

The predicted probabilities associated with each factor are mapped into points on a scale from 0 to 100. The presence or the level of each predictive factor is associated with a point system, allowing summing up the points for all the factors. The total points accumulated by the various covariates correspond to the predicted probability of incident MDR-TB. For example, for an individual with the characteristics of less than 60 years, a history of direct contact, CRT, unsuccessful treatment, excellent FCXE (such as 6 times), 3HRZES/6HRE, DPC (such as 3 months), and DPSC (such as 2 months) among individuals with CPTBT (see Table 5).

**Table 5:** Predicting an individual’s MDR-TB risk among individuals with CPTBT.

Risk factor	Value	Points
Age < 60 years	Yes	12.5
A history of direct contact	Yes	100
Passive mode of TB case finding	No	0
HIV infection	No	0
Completing re-treated TB treatment	Yes	4.0
Unsuccessful treatment	Yes	52.0
3HRZES/6HRE	Yes	18.0
Excellent FCXE	Yes	0
Duration of pulmonary cavities, months	3	12.0
Duration of positive sputum culture, months	2	24.0
Total points	222.5	
**Estimate of MDR-TB risk (%)**		
1-year probability of incident MDR-TB	71.5	
5-year probability of incident MDR-TB	83.5	
10-year probability of incident MDR-TB	93.5	

TB: tuberculosis; MDR-TB: multidrug-resistant tuberculosis; CPTBT: completing pulmonary TB treatment; HIV: human immunodeficiency virus; FCXE: frequencies of chest X-ray examination; H: isoniazid; R: rifampicin; Z: pyrazinamide; E: ethambutol; S: streptomycin.

## Discussion

Up to now, far too little attention has been paid to monitoring and managing the risk of incident MDR-TB among individuals with CPTBT, let alone developed a nomogram so as to comprehensively estimate an individualized risk of incident MDR-TB in individuals with CPTBT. In the present study, we performed a matched case–control study (1: 2 ratios) to explore the predictors of MDR-TB in individuals with CPTBT. According to results of this study, we constructed a comprehensive nomogram for providing a simple, precise and personalized prediction of incident MDR-TB among individuals with CPTBT. Our findings may provide more reliable evidences in developing prevention and control strategies of MDR-TB and guiding TB control executives’ decision-making (e.g., formulate the most effective surveillance, assessment and intervention measures for this population). We anticipate that these results will be useful in reducing the incidence of MDR-TB, the monitoring and management of individuals with CPTBT, and the design of clinical interventions for preventing MDR-TB.

The significance of this study is that it offers a few important features. First, this is the first nomogram for predicting MDR-TB risk in individuals with CPTBT that has collected enough risk factors to allow authentic forecast and assessment analyses. Second, in the validation analyses, whether internal (e.g., the C-index is 0.833 and 0.871 for the training and validation sets, respectively) or external, the comprehensive model outputted both sufficient accuracy and satisfied uniformity in predicting incident MDR-TB. Third, this tool would be easy to use in clinical practice, mainly because the applying of nomogram is very simple, convenient, and economical (i.e., an accurate evaluation is made by just using 10 dominant predictors of incident MDR-TB). Moreover, we observe that the running cost of this model is low, which lies primarily in developing a practice tool (i.e., a risk assessment scale) and incorporating this tool into the treatment information system of TB designated hospital. Fourth, comparing with the logistic regression model (e.g., it may not consider the impact of time effect for predicting the risk of MDR-TB), our study specifically considers estimating the risk of MDR-TB by using a semi-parametric model (i.e., Cox proportional hazard model) to maximize the Wald χ^2^ statistic.

In the present study, we found that 10 independent predictors were associated with the increased risk of MDR-TB in individuals with CPTBT. Similar results have been described in many previous studies on TB patients^3–6^. For example, regarding the unsuccessful treatment, it is still a key predictor for the control of incident MDR-TB. Thus, to monitor and manage the risk of MDR-TB, we not only focused on TB patients, but also concentrated on individuals with CPTBT.

Compared with risk factors of incident MDR-TB in TB cases, there were some different features on predictors of incident MDR-TB among individuals with CPTBT. A notable finding in this study was that a mild association was only found between CRT and incident MDR-TB. This is inconsistent with previous studies^3,28^, which suggested that re-treatment TB patients was significantly associated with MDR-TB risk. One possible explanation is that the difference is originated from a low susceptibility of drug resistance for re-treatment TB patients after the end of treatment^29^. To understand the cause further, the causal mechanism needs to be verified. Unlike a study conducted by Zhang et al.^30^, we observed that gender was not associated with MDR-TB risk. The present study also suggested that older age (≥ 60 years) did not correlate with the risk of MDR-TB. This had important public health implications for younger TB patients. Flora et al.^31^ reported that HIV infection was not strongly associated with MDR-TB risk. In this study, HIV infection was significantly associated with MDR-TB on the multivariate analysis. According to these data, we can infer that the early prediction and risk assessment of MDR-TB will be crucial among individuals with CPTBT. Resorting to this tool, we can comprehensively predict an individual with CPTBT’s personalized risk of MDR-TB.

In clinical practice, clinicians usually determine whether to treat TB patients according to the clinical, radiological and bacteriological features^32,33^. The excellent FCXE can early detect the relapse and ensure an immediate treatment in individuals with CPTBT. Thus, the excellent FCXE were advantageous to reducing the risk of MDR-TB among individuals with CPTBT. This finding, while preliminary, suggests that the government should take measures to guarantee the excellent FCXE of individuals with CPTBT in order to contain the epidemic of MDR-TB.

Interestingly, our study identified passive MCF (like physical examination, contact examination, and differential diagnosis of other diseases) as a strong risk factor for incident MDR-TB in individuals with CPTBT. The delayed diagnosis and treatment of TB, as we all know, potentially increased the risk for MDR-TB^12^. If the TB case finding was delayed, the TB case would develop into a serious TB leading to the course of treatment extended, it might become a risk factor associated with MDR-TB^12^. Thus, this finding has an important implication that the government should vigorously promote and develop the active finding mode of MDR-TB among individuals with CPTBT. Additionally, a TB control scheme including this nomogram should be formulated by our government.

It is worth mentioning that this study identified the association between 3HRZES/6HRE and the MDR-TB risk among individuals with CPTBT. According to the 2017 WHO guideline^34^, the category II regimen should no longer be prescribed during the treatment of re-treatment TB patients. Our finding might elucidate a key role of standardized TB treatment against incident MDR-TB and provide a strong evidence for the treatment of RPTPs. From the discussion, one may conclude that the DST should be performed to inform the choice of RPTPs’ TRs. Most notably, this study also observed that RPTPs were treated by using the TRs of 9-month, which were dramatically increased the risk of incident MDR-TB. This association may be attributed to the longer the time of exposure to anti-TB drugs, the greater the chance of occurrence of DR-TB^35^. To decrease the risk of MDR-TB, it is vital that standardized TRs are implemented by RPTPs.

Some researchers found a highly significant association between the contact with MDR-TB patient and incident MDR-TB^6,17,36^. Our study also suggested that a history of direct contact was one of the strongest independent predictors for incident MDR-TB in individuals with CPTBT. However, a prospective cohort study in Peru^37^ found that MDR-TB patients were less able to cause secondary disease in contacts, which might appear to conflict with the result of our study. After considering possible explanations of this discrepancy, our tentative suggestion is that the ethnic characteristic is associated with the risk estimate of MDR-TB^38^. This result implies that potential intervention measures like early detection of the high-risk population, early isolation and treatment of MDR-TB patient, and personal protective measures of susceptible persons, are urgently needed to curb the epidemic of MDR-TB among individuals with CPTBT.

Besides the novel identified predictors of incident MDR-TB, what the predominant finding in the present study was that we first integrated these existing predictors into an excellent risk prediction tool called nomogram^39^. According to this practical tool, we can comprehensively predict a personalized risk of incident MDR-TB among individuals with CPTBT.

Most importantly, the best way to interpret and apply these findings is not in terms of how the individual factors contribute to risk but how these parameters can be modified or improved to potentially decrease the incidence of MDR-TB^40^. Since the pathogenic mechanism of MDR-TB is still unclear, our findings and algorithm should be used to modify identified risk factors of MDR-TB in an effort to minimize morbidity. In terms of our findings, identifying the risk of incident MDR-TB for individuals with CPTBT may have an impact on the treatment, healthcare, surveillance, and management options of TB cases. In addition, the selection of TB patients who need additional treatment, or intensive surveillance and management remains controversial after completing treatment^41^. This clinic tool may be able to help physicians to solve such problems. Moreover, this nomogram can provide information in the design of clinical intervention, and guiding clinicians’ decision-making regarding the most effective intervention strategies among individuals with CPTBT. For example, according to this algorithm, an individual with CPTBT is found to be high-risk for incident MDR-TB. This finding has an important implication for developing the strategy of early intervention and management in the high-risk population of DR-TB. Overall, our results suggest that this nomogram may display the advanced public health concept of predictive, preventive, and personalized medicine^42^. This tool deserves to be further explored in future researches of clinic and public health. Considering these advantages of nomogram, our government should guide, support and foster the development of this tool. In addition, a control and prevention proposal including this tool for the risk of MDR-TB should be formulated by the government among individual with CPTBT.

Our study does have some limitations. First, our study is limited by the retrospective nature the data, which could suffer from recall bias and failure to incorporate some recognized prognostic parameters (e.g., the frequency or intensity of exposure). Second, potential confounders such as the mental health status of TB patients, TB drug quality and drug malabsorption could not be controlled. Third, we may not include them if MDR-TB cases did not go to a hospital. However, to reduce enrolment bias, we have retrieved and collected medical records (such as demographic, clinical and bacteriological data) of individuals with CPTBT from the non-local hospitals through the NTSS. Fourth, further efforts regarding prospective data collection and patient follow-up, wider geographic recruitment, and the incorporation of additional factors are encouraged to improve this tool. Despite these limitations, as we know, there are limited numbers of published data on the risk of incident MDR-TB in individuals with CPTBT. Therefore, this study could contribute information about the novel concept of predictive, preventive, and personalized medicine for incident MDR-TB.

## Conclusions

So far, unfortunately, we have failed to increase the clinician’s ability to properly predict an individual risk of MDR-TB among individuals with CPTBT. In the present study, we developed and validated a novel tool based on the status of less than 60 years, a history of direct contact, passive MCF, HIV infection, CRT, unsuccessful treatment, 3HRZES/6HRE, excellent FCXE, DPC, and DPSC, which predicted the probability of incident MDR-TB in individuals with CPTBT.

In conclusion, this tool can provide a vital role in counseling individuals with CPTBT and a novel strategy for the prevention and intervention of MDR-TB. In view of the high mortality and medical cost of MDR-TB cases, individuals with CPTBT are in urgent need of the early identifying of at-risk individuals and early intervening before the onset of MDR-TB.

## Data Availability

The datasets generated during and/or analyzed during the current study are available from the corresponding author on reasonable request.

## Abbreviations

MDR-TB: Multidrug-resistant tuberculosis
TB: Tuberculosis
DR-TB: Drug-resistant tuberculosis
CPTBT: Completing pulmonary TB treatment
CNDT: Completing newly diagnosed pulmonary TB treatment
CRT: Completing re-treated pulmonary TB treatment
NDPPs: Newly diagnosed pulmonary TB patients
RPTPs: Re-treated pulmonary TB patients
HDC: A history of direct contact
FCXE: Frequencies of chest X-ray examination
DST: Drug susceptibility testing
TRs: Treatment regimens
NTSS: National TB surveillance system
ROC: Receiver operating characteristic
AUC: Area under curve
HIV: Human immunodeficiency virus
TO: Treatment outcome
DPC: Duration of pulmonary cavities
DPSC: Duration of positive sputum culture
FDC: Fixed-dose combination
TIOFMV: Time interval from illness onset to the first medical visit
TIOLC: Time interval from illness onset to laboratory confirmation
H: Isoniazid
R: Rifampicin
Z: Pyrazinamide
E: Ethambutol
S: Streptomycin
WHO: World Health Organization
MTB: *Mycobacterium tuberculosis*
FMSM: Family members' management or self-management
CDM: Community doctor management
MCF: Mode of TB case finding
DAF: Duration of abnormal X-ray findings
FSC: Frequencies of sputum culture
DNSC: Duration of negative sputum culture
DWSC: Duration without sputum culture
FSS: Frequencies of sputum smear
DPSS: Duration of positive sputum smear
DNSS: Duration of negative sputum smear
DWSS: Duration without sputum smear
OR: Odds ratio
HR: Hazard ratio
CI: Confidence interval
